# La duplication rectale de l'adulte: une cause exceptionnelle de masse pelvienne

**DOI:** 10.11604/pamj.2015.22.346.8461

**Published:** 2015-12-10

**Authors:** Mohammed El Fahssi, Hicham Baba, Ahmed Bounaim, Abdelmounaim Ait Ali, Khalid Sair

**Affiliations:** 1 Service de Chirurgie Viscérale, Hôpital Militaire d'Instruction Mohammed V, Rabat, Maroc

**Keywords:** Duplication rectale, rectum, kyste rétrorectale, rectal duplication, rectum, retrorectal cyst

## Abstract

La duplication rectale est une anomalie constitutionnelle rare du tube digestif, elle représente 5% des duplications digestives son diagnostic se fait habituellement au tour de la période néonatale ou pendant les premières années de vie et ce n'est qu'exceptionnellement que son diagnostic reste méconnu jusqu’à l’âge adulte. Nous rapportant le cas d'une duplication rectale chez une femme de 41 ans se plaignant de douleurs pelviennes, l'examen clinique suivi des examens complémentaires ont suspecté le diagnostic la patiente est opérée par un abord antérieur isolé, permettant l'exérèse de la totalité de la masse kystique dont examen anatomo-pathologique confirme le diagnostic de duplication rectale kystique postérieure non communicante. L’évolution postopératoire est satisfaisante sans morbidité sur un recul de 20 mois.

## Introduction

Les duplications digestives sont des anomalies congénitales rares du tube digestif qui peuvent être uniques ou multiples et toucher n'importe quel segment depuis la bouche jusqu’à l'anus. La duplication iléale représente la localisation la plus fréquente, l'atteinte rectale est rare elle se produit dans 5% des cas. La duplication rectale peut prendre une forme kystique ou tubulaire communiquant ou non avec la lumière rectale. La forme kystique représente la forme la plus fréquente et elle est non communicante dans 90% des cas. C'est une pathologie congénitale qui est souvent diagnostiquée au tour de la période néonatale ou au cours des premières années de vie et C'est très rarement que le diagnostic reste méconnu jusqu’à l’âge adulte. Nous rapportant un cas de duplication rectale kystique diagnostiquée chez une femme de 41 ans ses particularités cliniques et radiologiques seront rapportées et sa prise en charge chirurgicale sera étayée.

## Patient et observation

**Figure 1:**
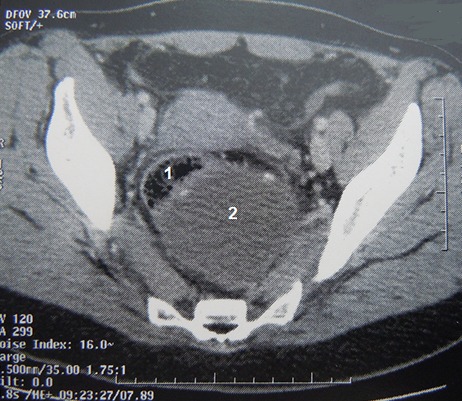
TDM abdomino-pelvienne en coupe transversale mettant en évidence le kyste retro-rectal de 9cm (2) comprimant le rectum (1)

**Figure 2:**
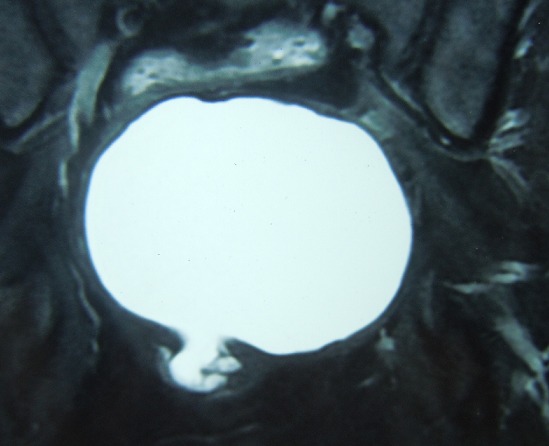
IRM pelvienne en coupe coronale en séquence pondérée T1 après injection de gadolinium mettant en évidence une grande masse kystique retro-rectale en hyper signal émettant par son pole inferieur un prolongement en doigt de gant vers le tiers inferieur du rectum

**Figure 3:**
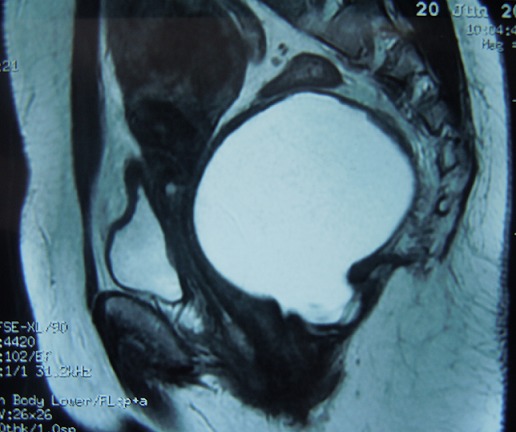
IRM pelvienne en coupe sagittale en séquence pondérée T2 Montrant une formation ovalaire présacré en hypersignal refoulant le rectum la vessie et le vagin en avant

Il s'agit d'une mère de 3 enfants âgée de 41 ans qui présente depuis un peu plus de 5 mois des douleurs de la fosse iliaque droite et de la région hypogastrique à type de torsion survenant par crises paroxystiques et d'intensité progressivement croissante accompagnés de ténesmes d’épreintes et de faux besoins, associée à des troubles du transit à type de constipation à raison d'une selle tous les 3 jours et sans notion de rectorragie. La patiente rapporte également des troubles urinaires à type de dysurie et de pollakiurie et des troubles menstruels à type de dysménorrhée et de ménorragie. Le tout évoluant dans un contexte d'apyrexie et de conservation de l’état général. L'examen clinique à l'admission trouve une patiente en bon état générale, aux conjonctives normalement colorées, apyrétique et dont l’état hémodynamique est stable. L'examen abdominal trouve une douleur et un empâtement à la palpation profonde de la fosse iliaque droite et de la région hypogastrique sans percevoir de masse évidente. Le toucher rectal trouve une masse rénitente postérieure à 5 cm de la marge anale, le sphincter est légèrement hypotonique, la marge anale est propre sans fissures,ni fistules, pas de trace de sang dans le doigtier. Le toucher vaginal perçoit la même masse postérieure. La recto-sigmoïdo-scopie objective un rétrécissement de la lumière du rectum en rapport avec une masse extra rectale bombant à la face postérieure refoulant et comprimant le rectum sans signes inflammatoires de la muqueuse en regard et sans défect pariétal. La tomodensitométrie abdominopelvienne a objectivé la présence d'une masse kystique rétrorectale de 9 cm de diamètre avec contact étroit avec la paroi rectale et extension le long des fosses ischio-anales avec des signes de compression extrinsèque du rectum (Figure 1). L'imagerie par résonance magnétique nucléaire (IRM) pelvienne objective la présence d'une formation kystique en hypersignal en T1et T2 à paroi rehaussée après injection de gadolinium mesurant 90x97x87mm refoulant le rectum en avant sans continuité avec celui-ci. Par son pole inferieur la masse kystique émet un prolongement en doigt de gant qui vient contracter des rapports étroits avec le tiers inferieur du rectum faisant suspecter le diagnostic d'une duplication digestive kystique rétrorectale non communicante (Figure 2, Figure 3).

**Figure 4:**
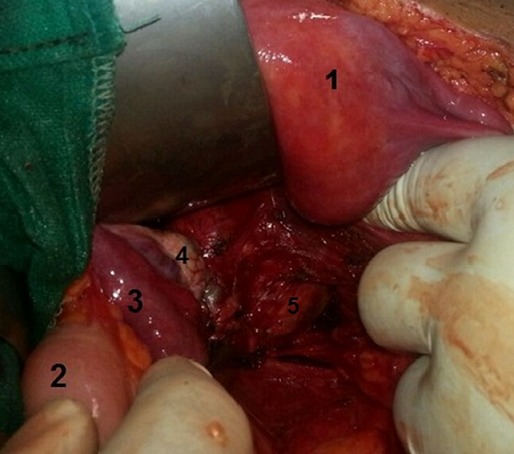
Aue opératoire montrant le pole supérieur de la masse kystique (5) au fond du champ opératoire après écartement du colon sigmoide (2) l'uterus (1) et ses annexes gauches: trompe gauche (3) et ovaire gauche (4).

**Figure 5:**
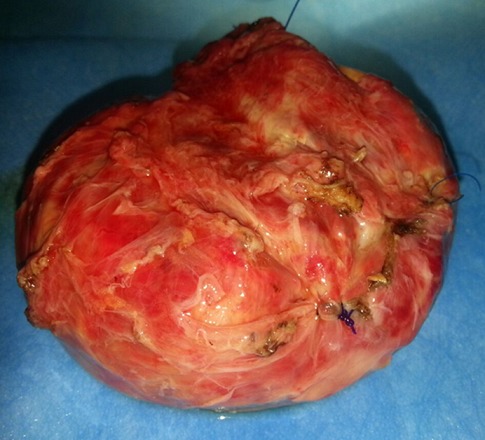
Aspect macroscopique de la pièce opératoire

**Figure 6:**
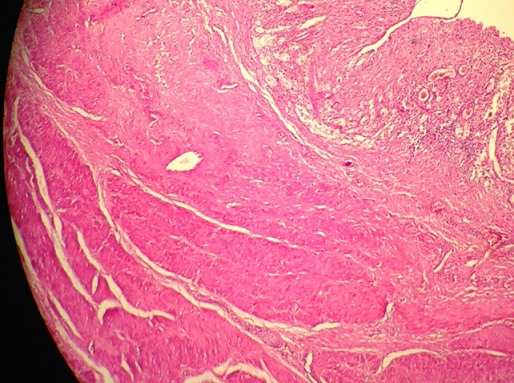
Aspect histologique de la duplication tapissée par un épithélium ulcéré par endroit parfois malpighien doublé par une couche musculaire lisse (HE X10)

Devant cette masse pelvienne kystique exerçant un effet de masse sur les organes de voisinage l'indication opératoire est retenue. Après une préparation colique la veille de l'intervention par une solution de polyéthylène glycol, L'intervention chirurgicale était menée sous anesthésie générale et en décubitus dorsal, par voie antérieure en réalisant une incision médiane sous ombilicale à cheval sur l'ombilic, après ouverture du péritoine et exposition, l'exploration trouve une grosse masse rénitente rétrorectale refoulant et comprimant le tube rectale en avant et à droite (Figure 4). La dissection est menée de proche en proche sur tous les contours du kyste. Un contact très intime, non dissécable, avec la paroi rectale étendue sur environ 2 cm a été noté sur le bord postéro-gauche du rectum. Cette zone de contact a été reséquée emportant une pastille de la paroi rectale pour permettre une exérèse complète de la lésion kystique (Figure 5). Lors de la dissection il y a eu une effraction de la paroi kyste avec issu d'un liquide brun chocolat, fluide non visqueux, ne contenant pas de débris tissulaire qui a été aspiré en totalité. La paroi rectale a été suturée. Un lavage abondant du pelvis a été réalisé au SS 9%°, puis drainage par deux Redons aspiratifs sortis dans le flanc gauche. Les suites opératoires étaient simples, premier lever le lendemain de l'intervention, reprise de l'alimentation au troisième jour postopératoire retrait des drains au quatrième jour et sortie le cinquième jour postopératoire. L’étude histologique de la pièce opératoire (Figure 6) a montré qu'il s'agit d'une duplication kystique du rectum sans signes de malignité avec une paroi tapissée d'un épithélium pavimenteux cylindrique parfois malpighien au niveau du pôle inferieur, ulcéré par endroit, reposant sur une épaisse couche musculaire lisse dont les fibres sont disposées dans deux sens différents. La patiente est revue à un mois puis tout les trois mois l’évolution est jugée très satisfaisante sans morbidité notable sur un recul de vingt mois.

## Discussion

La duplication rectale est une affection congénitale extrêmement rare dont la première description a été rapportée par Middeldorf en 1885, elle se présente sous deux formes kystique et tubulaire, la forme kystique étant présente dans plus de 90% des cas [1]. Il s'agit d'une malformation congénitale donc présente à la naissance qui reste asymptomatique jusqu´à ce que le volume du liquide intrakystique aura atteint un niveau optimum pour causer des manifestation obstructives et compressives sur les organes voisins ou la survenu d'une infection intrakystique qui se produit plus volontiers dans les formes communicantes. Ce qui explique que malgré la majorité des cas diagnostiqués au cours de l'enfance il existe des cas qui restent quiescents et ne deviennent symptomatiques que plus tard à l’âge adulte [2]. Les deux sexes sont touchés mais une prédominance féminine est notée [1, 3]. L’éthiopathogénie de la duplication rectale reste controversée Plusieurs théories ont été avancées pour tenter d'expliquer la genèse des duplications mais trois restent les plus retenues,théorie de la notochordodysraphie par altération de l'isolement de la notochorde du tube digestif à la 3eme semaine de l'embryogenèse est la plus plausible puisqu'elle explique aussi la position mésentérique dorsale des duplications,la fréquence des hétérotopies muqueuses dérivées des cellules multipotentes de l'intestin primitif et l'association avec d'autres malformations embryonnaires. La théorie de l'erreur de reperméabilisation canalaire et la théorie du déterminisme vasculaire Sont deux autres théories possibles [1, 4]. La duplication rectale se présente sous forme kystique dans 95% des cas dans où elle est habituellement non communicante et postérieure ou latérale [2, 5]. La forme tubulaire est rare retrouvée dans 5% des cas réalise un véritable dédoublement du tube digestif en canon de fusil souvent communicante par son extrémité distale ou proximale ou par les deux extrémités. Leur paroi épaisse peut s´amincir sous l´effet de la pression des secrétions accumulées, pouvant même entraîner une nécrose pariétale, voire une perforation intra-digestive ou en péritoine libre. Leur contenu est un liquide clair, séreux ou coloré, souvent mucoïde, dont le PH varie avec la nature de la muqueuse. Le liquide peut être hémorragique ou noirâtre lorsqu´il y a de la muqueuse gastrique. Lorsque la duplication est communicante son contenue est alors digestif [1, 6]. Ladd and Gross en 1940 proposent les critères de définition d'une duplication digestive [1, 5] à savoir: la contiguité et l'adhérence au segment digestif correspondant; l'existence d'une couche musculaire lisse au sein de la paroi ( fibres musculaires dans deux sens différents); un revêtement muqueux tapissant intérieur de la paroi.

Souvent asymptomatique découverte alors de façon fortuite lors d'un examen systématique La Symptomatologie clinique révélatrice est le plus souvent d'installation progressive faite de douleurs pelviennes,de constipation, de ténesmes et de faux besoins,des complications hémorragiques peuvent être inaugurales en rapport soit avec une nécrose ischémique par compression ou avec l'existence d'une hétérotopie muqueuse gastrique. La palpation d'une masse abdomino-pelvienne est rare, Le toucher rectal peut percevoir une masse rénitente [7]. La rectoscopie peut visualiser l'effet de masse de la duplication rétrécissant la lumière rectale et identifie un éventuel orifice de communication. La radiographie de l'abdomen sans préparation peut montrer un refoulement des structures digestives par une opacité de tonalité hydrique contenant éventuellement de l'air dans les formes communicantes. Lavement baryté peut montrer une opacification directe de la duplication dans les formes communicantes ou mettre en évidence une image de compression extrinsèque. L’échographie abdominale est un examen non invasif d'un apport capital permettant de suspecter le diagnostic de la duplication rectale devant une masse kystique qui présente une paroi similaire à celle d'une anse digestive organisée en deux couches hyperéchogène interne correspondant à la couche muqueuse et hypoéchogène externe correspondant à la musculeuse. Le contenu kystique peut être liquidien anéchogène avec un renforcement postérieur en cas de contenu mucoïde ou séreux, ou échogène en cas d'hémorragie intrakystique, d'infection ou si il existe une communication avec la lumière rectale [1, 8]. Elle peut également objectiver les mouvements péristaltiques au sein de la masse [8] évocateurs de la nature digestive de la masse. La tomodensitométrie abdomino-pelvienne montre une masse kystique hypodense contiguë à la paroi rectale prenant le contraste comme une structure digestive, elle précise son extension et ses rapports. La présence de remaniement hémorragique ou infectieux intra kystique peut réaliser l'aspect d'une masse tissulaire ou d'une tumeur nécrosée. Elle permet également de rechercher les autres malformations associées vertébrales et génito-urinaires qui sont présents dans 20% des cas [7–10].

L'imagerie par résonance magnétique pelvienne permet d'identifier les différentes couches de la paroi de la duplication posant le diagnostic et contribuant ainsi au diagnostic différentiel avec les autres masses pré sacrées rétro rectales. La muqueuse et la sous muqueuse paraissent en hypersignal et la musculaire muqueuse et la musculeuse en hypo signal. Elle permet aussi de donner une meilleure étude des rapports anatomiques de la lésion avec les organes de voisinage ce qui est très utile pour guider le traitement chirurgical [8, 11]. Echo-endoscopie transrectale est une méthode d'imagerie très sensible (80 à 85%), très usuelle, très facile à utiliser non invasive, apporte les mêmes renseignements que l'IRM et elle est techniquement plus rapide [9], mais limitée par la présence d'air au sein des duplications communicantes rendant plus utile l'exploration par l'IRM [11]. La duplication rectale peut être découverte au stade de complications qui peuvent être d'ordres mécaniques par compression du rectum, des voies génito-urinaires et des plexus nerveux avoisinants,d'ordres infectieuses par rupture de la duplication en péritoine libre ou dans un organe de voisinage ou d'ordres hémorragiques dues à l'existence de foyers d'hétérotopie de muqueuse gastrique. Mais la complication la plus redoutable reste la dégénérescence maligne. Décrite initialement par Ballantyne en 1932 elle touche 12% des duplications rectales de l'adulte, l'adénocarcinome étant la forme la plus fréquente [1, 6, 4]. Le traitement de ses duplications rectales ne peut être que chirurgical il dépend de la topographie et de l'aspect anatomique de la duplication le geste à réaliser peut être une exérèse totale simple si les rapports de la duplication le permettent. Dans les cas où les rapports sont très intimes, et Chaque fois que le geste s'avère sacrifiant on peut avoir recours à l’énucléation ou l'exérèse subtotale avec électrocoagulation ou exérèse de la muqueuse du fond de coquetier respectant le segment dupliqué.

Différentes voies d'abord sont proposés: la voie antérieure isolée, la voie combinée par abord antérieur et postérieur, et la voie postérieure isolée. La voie d'abord laparoscopique ou la microchirurgie trans-anale endoscopique TME est actuellement recommandée pour les duplications de petite taille [1, 3, 10]. Le choix de la voie d'abord est discuté en fonction de la taille, de la hauteur de la duplication par rapport aux pièces sacrées, du mode d'insertion de la duplication sur le rectum et de la survenue d’éventuelles complications [12]. Le toucher rectal aide au choix de la voie d'abord; si le bord supérieur de la duplication est palpé, l'abord sera postérieur. Pour les duplications situées au dessus de S3 ou de diamètre inferieur à 4cm la voie antérieure est recommandée. Pour les duplications situées au dessous de S3 et de diamètre inferieure à 4cm la voie postérieure est privilégiée par une voie trans-coccygiennne, trans-sacrée de Kraske, voie périnéale,voie retro-ano-rectale,ou par voie inter-sphinctérienne. Pour les duplications de diamètre plus important la voie abdominale isolée ou combinée à une voie postérieure est utilisée. Le pronostic dépend surtout de la précocité du diagnostic et de la prise en charge thérapeutique. il est généralement favorable en dehors de certaines complications dont la transformation maligne reste la plus redoutable.

## Conclusion

La localisation rectale des duplications digestives est une entité rare, voire exceptionnelle chez l'adulte. Si la définition anatomopathologique de la duplication est unanimement admise, sa pathogénie reste très discutée. Le diagnostic positif est suspecté sur la clinique et fortement évoqué sur les examens radiologiques Mais seul l'examen anatomopathologique est capable de confirmer le diagnostic. Le traitement de la duplication rectale ne peut être que chirurgical et l'exérèse complète du kyste et la technique de choix pour éviter les complications surtout la transformation maligne. La voie d'abord dépend de la localisation, le type, la taille et la présence ou non de complications. En définitive, en dehors des formes compliquées, le pronostic de la duplication rectale reste excellent lorsqu'elle est diagnostiquée et traitée à temps.
